# Tiamulin and monensin intoxication in commercial brown pullets

**DOI:** 10.1007/s11259-025-10771-3

**Published:** 2025-05-20

**Authors:** Álex Gómez, Sergio Villanueva-Saz, Antonio Fernández, Estela Pérez, Lluís Luján, Diego Cortés

**Affiliations:** 1 Animal Pathology Department, Zaragoza University, C. de Miguel Servet, 177, Zaragoza, 50013 Spain; 2 Instituto Agroalimentario de Aragón-IA2, Zaragoza University, Zaragoza, Spain; 3 Ibérica de Tecnología Avícola, IBERTEC S.A.U, Valladolid, Spain

**Keywords:** Tiamulin, Monensin, Intoxication, Pullets

## Abstract

Tiamulin and monensin intoxication has been widely reported in broilers and many other domestic animals, causing muscular dystrophy. This study reports an outbreak of subacute tiamulin-monensin toxicosis in commercial brown pullets and provides a detailed clinicopathological description. The outbreak occurred on a floor-rearing farm in Spain of 21,044 Lohmann Brown pullets (11–16 weeks old) after monensin (120 ppm) was administered in feed and tiamulin (150 ppm) in drinking water. Within 24 h after tiamulin treatment, feed and water consumption declined drastically, and mortality increased progressively, reaching a peak of 1.75%. Cumulative mortality over a 13-day period was 4.3%. Affected pullets (8%) exhibited marked apathy, muscle weakness, flaccid paralysis, and prostration. Necropsy of 18 naturally dead pullets revealed gross myocardial (72%) and vastus intermedius (100%) muscle pallor, with histopathological examination confirming monophasic myocardial and myocyte degeneration and necrosis (100%). Fibrosis and granulomatous infiltration surrounding and infiltrating affected muscle fibers were observed in a low number of animals (11%). These findings suggest that tiamulin enhances monensin toxicity through biochemical interactions, indicating that the combination of 150 ppm tiamulin and 120 ppm monensin is highly toxic to pullets. This highlights the need for careful administration in poultry production. Twenty-four hours after tiamulin-monensin withdrawal, feed and water consumption increased and mortality decreased. Tiamulin-monensin toxicosis should be considered in the differential diagnosis of cardiomuscular disorders in pullets and layers.

**Supplementary Information:**

The online version contains supplementary material available at 10.1007/s11259-025-10771-3.

## Introduction

Tiamulin is a semi-synthetic diterpene antibiotic widely used in poultry medicine to treat bacterial diseases such as mycoplasmosis and mrachyspirosis (Islam et al. 2009). When administered alone, tiamulin presents a low risk of toxicity; however, when co-administered with other drugs, particularly monovalent polyether ionophore anticoccidials such as monensin, a strong pharmacological interaction occurs, leading to intoxication characterized by severe clinical signs (Cooper and Valentina 2016). The interaction between tiamulin and monensin is a well-documented phenomenon in chickens and turkeys, reported for decades (Islam et al. 2009).

Tiamulin acts as a direct inhibitor of cytochrome P4503 A (CYP3 A) enzymes, which are key isoforms of the cytochrome P450 family involved in drug metabolism. Consequently, CYP3 A inhibition prevents the hepatic O-demethylation of monensin, impairing ionophore metabolism and leading to increased accumulation of monensin in the liver and bloodstream (Szucs et al. 2004). Ionophores facilitate cation transport across plasma membranes, and their primary toxic mechanism involves disruption of Na^+^/K^+^-ATPase and Ca^2+^-ATPase enzyme activity in muscle cells. This disruption results in intracellular calcium overload and inhibition of mitochondria-mediated oxidative phosphorylation (Caló et al. 2002; Ekinci et al. 2023). The myocardium and skeletal muscles are particularly susceptible, likely due to their high metabolic activity (Novilla 2018). The median lethal dose (LD_50_) for acute toxicity varies across species. In chickens, the LD_50_ of monensin ranges from 200 to 214 ppm (Ekinci et al. 2023). Furthermore, monensin at 100 ppm, when administered concurrently with tiamulin at 270 ppm in drinking water, has been shown to induce severe muscular pathology in broilers (Badiola et al. 1994).

The clinical signs of tiamulin-monensin toxicity are similar to those observed with monensin overdose alone (Szucs et al. 2004). These signs arise due to severe disturbances in ion transport between myocytes/cardiomyocytes and the intercellular space and include reduced feed intake, poor weight gain, muscle weakness, and flaccid paralysis. Affected birds typically exhibit sternal recumbency with an extended neck and legs (Fulton 2020). Mortality rates are highly variable and depend on several factors, including the tiamulin-monensin dose, duration of monensin exposure, and the age of the chickens (Sandercock and Mitchell 2004; Rath et al. 1998). Additionally, monensin at 100 ppm in feed has been reported to adversely affect the fertility of broiler-breeder hens (Jones et al. 1990).

Pathological lesions associated with monensin toxicity are primarily linked to elevated extracellular potassium and increased intracellular (intramitochondrial) calcium levels, leading to mitochondrial dysfunction, ATP depletion, cellular oedema, myocyte degeneration, and necrosis (Madej et al. 1993; Islam et al. 2009). The nature of the lesions varies depending on the chronicity of intoxication. In acute toxicosis (48 h), no gross lesions may be observed (Chalmer 1981). In subacute toxicosis (days to weeks), the heart and skeletal muscles exhibit scattered areas of hyalinization, muscle necrosis, and myofiber degeneration. Subchronic toxicosis is characterized by opaque fibrin plaques on the epicardium, coronary fat hemorrhages, and reduced liver weight (Fulton 2020). In these later stages, muscular fibrosis and heterophils, macrophages, and occasional lymphocytes are found surrounding and infiltrating necrotic muscle fibers.

To the authors’ knowledge, this is the first report describing subacute tiamulin-monensin toxicosis in commercial Lohmann Brown pullets, providing valuable insights into its clinical progression and pathological manifestations.

## Materials and methods

### Case history

This outbreak was observed in a floor-rearing farm of 21,044 commercial Lohmann Brown pullets (11 to 16-weeks-old) from Vizcaya (Spain) with a diet based on mash feed. Animals were vaccinated (Paracox 5^®^) against coccidiosis at the hatchery. This vaccine does not include *Eimeria necatrix* or *E. maxima* in its formulation. Therefore, at 11.5 weeks of age, monensin (Elancoban G200^®^, 200 g/kg of feed) was preventively incorporated into the feed at a dose of 0.6 kg/ton (120 ppm). Two days after initiating monensin treatment, elevated environmental ammonia levels resulting from poor ventilation management caused respiratory and ocular signs. Tiamulin (Hidromutin^®^ 125 mg/ml) was administered in the drinking water at a dose of 1.2 mL/L (150 ppm) for five days to prevent secondary infections. Monensin administration was removed at 9 days post-treatment (dpt) and animals were supplemented with an aminoacidic-multivitamin complex (Hidro Rex Vital Aminoácidos^®^) at a dose of 1 ml/L.

## Pathological study

Eighteen naturally died animals were selected to perform a detailed necropsy at 8 dpt. Heart, striated muscle (vastus intermedius and pectoral), bone marrow, kidneys, lungs, liver, central nervous system and eyes were sampled and fixed in 10% neutral-buffered formalin. Samples were remitted to the University of Zaragoza and embedded in paraffin, and 4 μm-thick sections were stained with hematoxylin and eosin (HE) for histopathological study.

## Results

### Clinical findings

Twenty-four hours after first tiamulin administration, feed and water consumption drastically decreased, and mortality progressively increased, peaking at 8 dpt (Table 1). In farm, approximately 8% of the animals presented lethargy, muscle weakness, feather ruffling, neck retraction, lack of response to stimuli, and prostration (Supplementary material S1). After monensin withdrawal (9 dpt), both consumptions significantly increased, and mortality gradually declined (Table 1). Cumulative mortality over a 13-day period was 4.3% (*n* = 903/21,044). From 18 selected animals for pathological study, 38.9% (*n* = 7/18) presented sternal recumbency with an extended neck and legs, as well as, respiratory dyspnea and 61.1% (*n* = 11/18) showed apathy, feather ruffling, neck retraction, refusal to consume feed and water and very mild response to stimuli.

**Table 1:** Daily productivity data after monensin treatment (dpt). Monensin was applied at 0 Dpt and tiamulin was administered at 2–6 Dpt. Monensin was retired at 9 Dpt

dpt	Nº dead animals	Mortality (%)	Cumulative mortality (%)	Individual feed consumption (g/animal/week)	Individual water consumption (mL/animal/week)
0	2	0.01	0.01	71	99
1	12	0.06	0.07	72	101
2	5	0.02	0.09	70	80
3	7	0.03	0.12	52	29
4	14	0.07	0.19	58	45
5	8	0.04	0.23	62	47
6	20	0.1	0.32	56	56
7	195	0.94	1.26	49	110
8	362	1.75	3.01	50	102
9	100	0.48	3.49	59	107
10	45	0.21	3.7	72	108
11	56	0.27	3.97	80	108
12	46	0.22	4.19	88	110
13	33	0.16	4.34	82	111

## Pathological findings

**Fig. 1:**
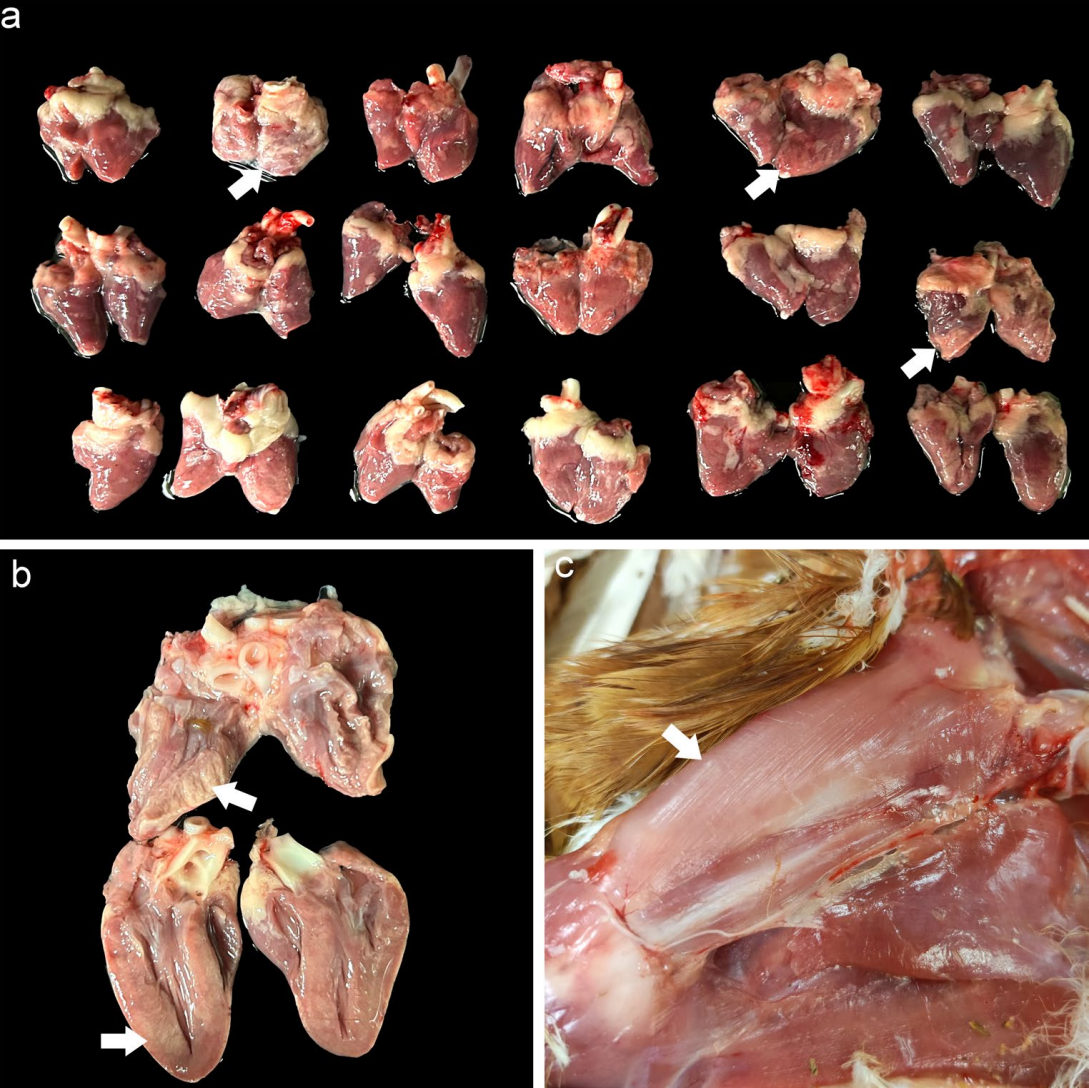
Eighteen commercial brown pullets affected with subacute tiamulin-monensin toxicosis. **a**) Some hearts present multifocal to coalescence pallor of myocardium (arrows). **b**) Myocardial pallor affect the entire thickness of the myocardium. **c**) Vastus intermedius muscles of an affected hen, showing diffuse pallor

Macroscopically, 72% (*n* = 13/18) of the animals exhibited multifocal to coalescing myocardial pallor (Fig. 1a and b), while 100% (*n* = 18/18) and 17% (*n* = 3/18) showed diffuse pallor of the vastus intermedius and pectoral musculature, respectively (Fig. 1c). Additionally, 28% (*n* = 5/18) of the hens displayed mild to moderate pulmonary congestion and oedema, as well as hepatic congestion. No gross lesions were observed in other tissues.

**Fig. 2:**
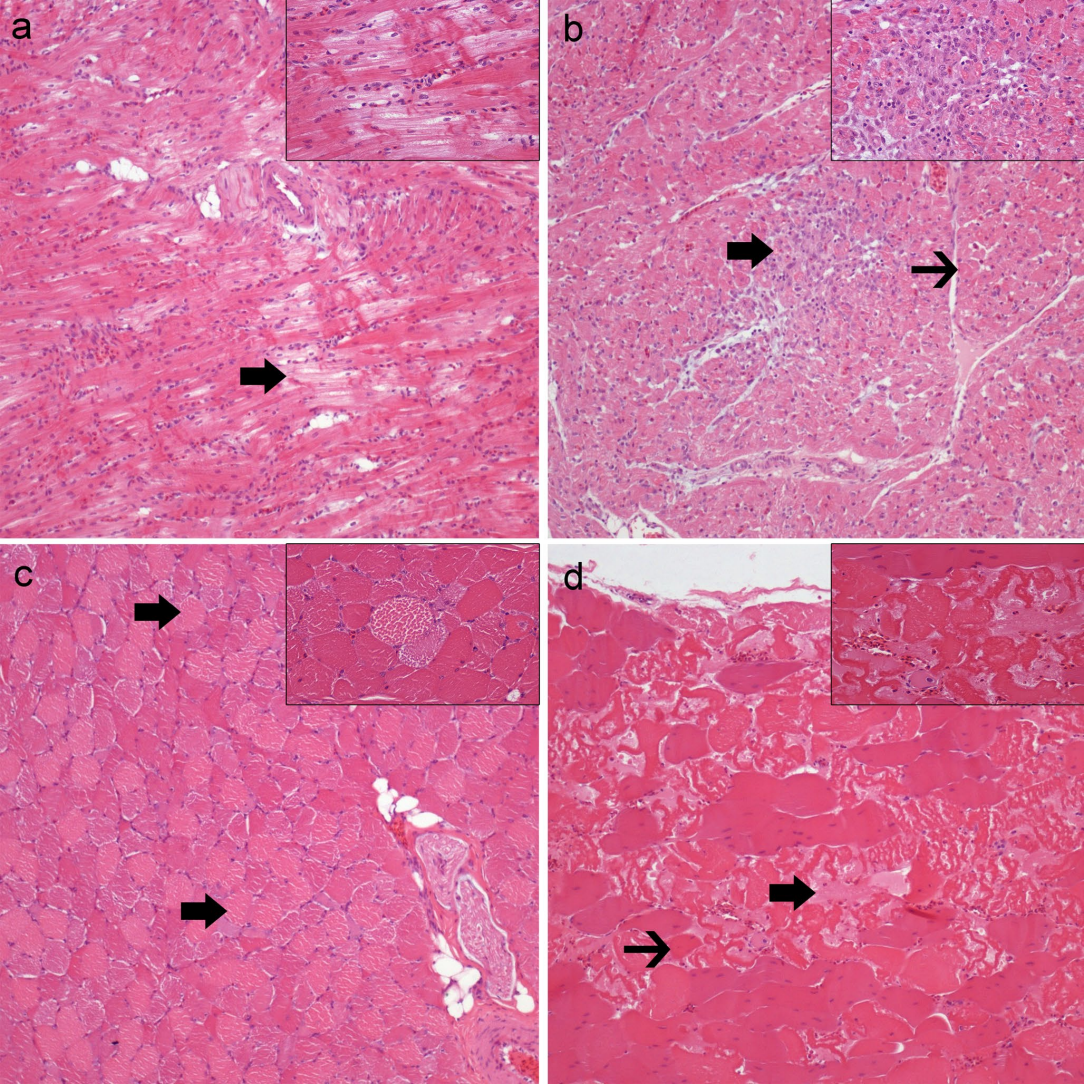
Histopathological features of the heart, vastus intermedius and pectoral muscle from 18 commercial brown pullets affected with subacute tiamulin-monensin toxicosis. Haematoxylin-eosin. **a**). Degenerated myocardiocytes (arrow) characterized by pale eosinophilic cytoplasm. Inset: affected myocardiocytes show swollen, vacuolated sarcoplasm and loss of cross-striations. **b**) Degenerated cardiomyocytes are surrounded and infiltrated by macrophages and focally replaced by fibrous tissue (arrow). Inset: higher magnification of fibrous and granulomatous foci effacing and replacing degenerated cardiomyocytes. **c**) Degenerated myocytes of vastus intermedius present pale eosinophilic sarcoplasm. Inset: affected myocytes show a marked intracytoplasmic oedema and loss of cross striations. **d**) Degenerated (arrow) and necrotic (thin arrow) myocytes of the pectoral muscle, admixed with a moderate intercellular oedema. Inset: Affected myocytes are characterized by a complete loss of cross-striations and nuclei

Microscopically, all animals (100%, *n* = 18/18) presented multifocal monophasic myocardial degeneration and necrosis. Degenerated cardiomyocytes exhibited swollen, vacuolated sarcoplasm and loss of cross-striations (Fig. 2a). Necrotic cardiomyocytes were characterized by hypereosinophilic, fragmented sarcoplasm with disorganized or hyalinized myofibrils and nuclear pyknosis (Fig. 2b). Specifically, 28% (*n* = 5/18) of the hens showed diffuse and severe myocardial degeneration/necrosis, while 72% (*n* = 13/18) exhibited multifocal mild to moderate myocardial degeneration/necrosis. Only 11% (*n* = 2/18) of the animals presented multifocal mild granulomatous inflammatory infiltrate and fibrosis associated with necrotic myocardial areas (Fig. 2b). In 100% (*n* = 18/18) and 17% (*n* = 3/18) of the cases, the vastus intermedius muscle and pectoral displayed multifocal moderate degeneration/necrosis, respectively, exhibiting the same histopathological features aforementioned (Fig. 2c and d). No inflammatory infiltrates or fibrosis were observed in the skeletal muscle. Moreover, in 28% (*n* = 5/18) of the hens a diffuse and mild alveolar oedema with a moderate pulmonary hyperemia and diffuse, moderate sinusoidal hyperemia were observed. No microscopic lesions were detected in other tissues.

## Discussion

This study represents a report of subacute tiamulin-monensin toxicosis in commercial brown pullets and provides a detailed clinicopathological description of the outbreak. The definitive etiological diagnosis was based on clinical history, histopathological examination and the response to treatment withdrawal. In cases of subacute tiamulin-monensin toxicosis, clinical signs may be mistaken for neurological disorders such as highly pathogenic avian influenza, Newcastle disease, Marek´s disease or avian encephalomyelitis (Fulton 2020). Therefore, in such cases, it is essential to determine the administered doses of tiamulin and monensin in the feed and perform a macroscopic and microscopic examination of the central nervous system, cardiac and skeletal muscle. Although not conducted in this study, measuring blood and muscular concentrations of the suspected drugs and performing biochemical analyses to assess muscular, hepatic and renal enzyme levels could be useful for diagnosing and tracking this type of intoxication. Additionally, other nutritional deficiencies, such as selenium and vitamin E deficiency, or bacterial infections, such as botulism, should be ruled out through a comprehensive analysis of the feed formulation and safety.

In this study, subacute tiamulin-monensin toxicosis induced a progressive increase in mortality (1.26–3.27%) 24 h after treatment with tiamulin, which was higher than the mortality rates previously reported in broilers (Badiola et al. 1994). However, the severity of tiamulin-monensin toxicosis depends on factors such as the age of the animals, as well as the administered doses (Ekinci et al. 2023). Therefore, the findings of this study suggest that 150 ppm of tiamulin and 120 ppm of monensin are highly toxic to pullets. The recommended monensin dose in chickens ranges from 100 to 125 ppm; however, 100 ppm of monensin has been shown to significantly impair growth in chickens (Keshavarz and Mcdougald 1982). Furthermore, 100 ppm of monensin in feed has been associated with reduced fertility in broiler-breeder hens, although no other clinical signs were observed (Jones et al. 1990). Other studies have reported that 270 ppm of tiamulin and 100 ppm of monensin were toxic to broilers (Badiola et al. 1994). The administration of tiamulin may lower the toxic threshold of monensin due to biochemical interactions between these compounds (Szucs et al. 2004). Therefore, tiamulin and monensin should not be administered together in pullets or laying hens to prevent toxic effects.

The clinical signs and lesions observed in this study closely resembled those previously described in broiler intoxications (Fulton 2020). Only the myocardium and skeletal muscle were affected, consistent with findings in younger chickens (Ekinci et al. 2023). Degeneration and necrosis of cardiomyocytes and myocytes were observed, with considerable variability among affected animals. Gross lesions in the heart were present in 72% of the cases, whereas 100% of the pullets exhibited muscular pallor in the quadriceps. These results suggest that vastus intermedius and pectoral muscles are more commonly affected than cardiac muscle. However, due to the high variability in gross lesion presentation, myocardium and skeletal muscle samples should be collected for histopathological examination in cases where tiamulin-monensin toxicosis is suspected.

Following the withdrawal of monensin treatment, feed-water consumption increased and mortality decreased. However, the elevated cumulative mortality suggests that severely affected animals were unable to overcome their condition. The remaining animals may or may not regain productivity, depending on the extent of muscle damage (Ekinci et al. 2023). Interestingly, the complete withdrawal of both treatments demonstrated an immediate improvement in the mortality rate. Therefore, the diagnosis of an acute or subacute toxicosis may facilitate a rapid resolution of the outbreak, allowing for the progressive recovery. However, further studies are needed to assess the long-term impact of tiamulin-monensin intoxication on a flock of pullets or hens.

The definitive diagnosis of tiamulin-monensin toxicosis was based on clinical history, histopathological findings and the response to treatment withdrawal. This study suggests that 150 ppm of tiamulin and 120 ppm of monensin may be toxic to commercial pullets and laying hens. Therefore, tiamulin-monensin intoxication should be considered in the differential diagnosis of cardiomuscular disorders in commercial poultry.

## Electronic supplementary material

Below is the link to the electronic supplementary material.

Supplementary Material 1

## Data Availability

No datasets were generated or analysed during the current study.
